# Antigenic sites on the H_N_ domain of botulinum neurotoxin A stimulate protective antibody responses against active toxin

**DOI:** 10.1038/srep15776

**Published:** 2015-10-28

**Authors:** B. Vijayalakshmi Ayyar, Rajeev B. Tajhya, Christine Beeton, M. Zouhair Atassi

**Affiliations:** 1 Department of Biochemistry and Molecular Biology, Baylor College of Medicine, Houston, Texas 77030, USA; 2 Department of Molecular Physiology and Biophysics, Baylor College of Medicine, Houston, Texas 77030, USA; 3 Department of Pathology and Immunology, Baylor College of Medicine, Houston, Texas 77030, USA

## Abstract

Botulinum neurotoxins (BoNTs) are the most toxic substances known. BoNT intoxicates cells in a highly programmed fashion initiated by binding to the cell surface, internalization and enzymatic cleavage of substrate, thus, inhibiting synaptic exocytosis. Over the past two decades, immunological significance of BoNT/A C-terminal heavy chain (H_C_) and light chain (L_C_) domains were investigated extensively leading to important findings. In the current work, we explored the significance of BoNT/A heavy chain N-terminal (H_N_) region as a vaccine candidate. Mice were immunized with recombinant H_N_519–845 generating antibodies (Abs) that were found to be protective against lethal dose of BoNT/A. Immuno-dominant regions of H_N_519–845 were identified and individually investigated for antibody response along with synthetic peptides within those regions, using *in vivo* protection assays against BoNT/A. Results were confirmed by patch-clamp analysis where anti-H_N_ antibodies were studied for the ability to block toxin-induced channel formation. This data strongly indicated that H_N_519–593 is an important region in generating protective antibodies and should be valuable in a vaccine design. These results are the first to describe and dissect the protective activity of the BoNT/A H_N_ domain.

Botulinum neurotoxin is a protein toxin produced by the anaerobic bacterium *Clostridium botulinum.* It is the most lethal toxin known. Eight serological serotypes (A through H) along with a number of subtypes of these serotypes have so far been identified. The toxin is composed of two subunits, a heavy (H) chain (molecular weight 100-kDa) and a light (L) chain (molecular weight 50-kDa) linked together by a disulfide bond. The H chain enables the toxin to bind to the neuronal cell membrane, after which the toxin enters the cell by endocytosis and causes paralysis. Inside the cell, the L chain, which is a Zn-endopeptidase, is unconstrained in the endocytotic vesicles and is set free in the cytoplasm where it cleaves the SNARE protein which is required for vesicle fusion, necessary for neurotransmitter (acetylcholine) release at the neuromuscular junction. Thus, the toxin interferes with passage of nerve impulses.

The binding of the toxin to the cell membrane has been attributed to a binding site located in the C-terminal (H_C_) domain of the H chain, whereas the translocation of L chain into the cell is attributed to the channel formation by N-terminal (H_N_) domain of the H chain. Considerable data has supported the presence of a binding site on the H_C_ domain. However, Maruta *et al.*^1^, using synthetic 19-residue overlapping peptides spanning the entire H chain of botulinum neurotoxin serotype A (BoNT/A), found regions within peptides N21 (residues 729–747), N23 (757–775) and N26 (799–817), on BoNT/A H_N_ that were involved in the binding of the toxin to mouse brain synaptosomes (SNPs). Very recently, we expressed H_N_ peptide 729–845 and found that this H_N_ region was able to bind directly to SNPs and also inhibit substantially BoNT/A binding to SNPs^2^. Most significantly, it completely protected mice against a lethal dose (1.05 × LD_100_) of BoNT/A leading to conclusion that H_N_729–845, and by extension the H_N_ domain, is fully pre-set to bind to neuronal cells and in the free state can even compete with the toxin for binding to the neuronal cell.

The antigenic sites on a protein reside at surface regions of the molecule^3^,^4^ which very frequently superimpose or overlap with the regions that are involved in other biological activities^5^,^6^,^7^,^8^. This would indicate that immunization with BoNT/A should stimulate antibodies (Abs) that could interfere with the toxic action of BoNT/A. Also the H_N_ domain of BoNT/A has a distinct mode of folding that appears to be dependent mostly on internal domain interactions and therefore immunization with this domain could give Abs that would be similar to the anti-toxin Abs directed against this region.

In the current studies, we cloned a peptide corresponding to BoNT/A residues 519–845, which comprised almost the entire H_N_ domain, and a shorter (75 residue) segment corresponding to sequence 519–593. We generated Abs against both peptides and against previously described peptide H_N_729–845^2^ and mapped the specificity of each of these three anti-H_N_ Abs by synthetic peptides, spanning the recombinant H_N_ region. Based on the Ab mapping data, we investigated the abilities of these Abs to inhibit *in vitro* channel formation by BoNT/A and to exhibit *in vivo* protection against lethal doses of active toxin.

## Results

### H_N_519–845 expression and purification

**Figure 1:**
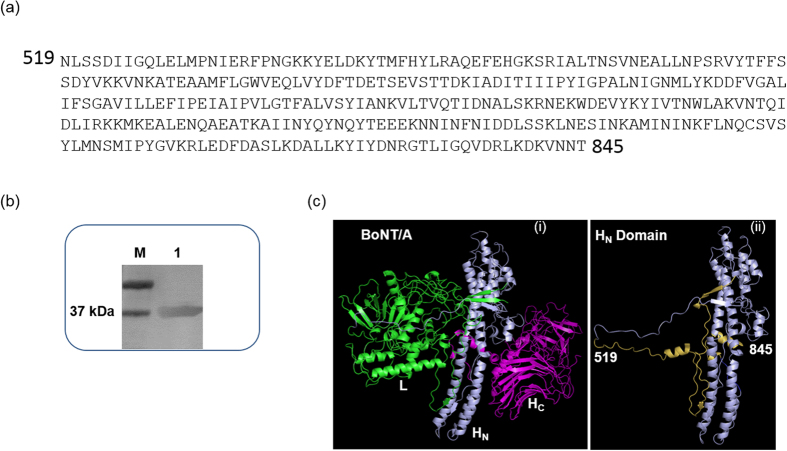
Structural characterization of BoNT/A peptide H_N_519-845. (**a**) Amino acid sequence of the recombinant peptide H_N_519–845 prepared in the present work. The peptide consists partial belt sequence (519–546) along with two α-helical loops containing the full transmembrane region (618–661). (**b**) SDS-PAGE analysis of purified peptide H_N_519–845 showed a high yield of purified peptide (>95%) obtained from the inclusion bodies, purified using denaturing conditions. The peptide was refolded using 0.5% (w/v) sodium lauryl sarcosine. Lane M, Bio-Rad Precision Plus Protein^TM^ Dual Xtra Standard; Lane 1, purified H_N_519–845. (**c**) (i) The 3-dimensional structure of whole BoNT/A with individual domains marked (ii) the H_N_ domain of BoNT/A with yellow region showing the part of belt region expressed in H_N_519–845.

H_N_519–845 was expressed successfully in *E. coli* BL21(DE3)pLysS cells providing 1 mg/ml of 90–95% of pure H_N_519–845 per liter of bacterial culture (Fig. 1A,B). The peptide was further characterized by CD spectroscopy analyses (data not shown). Secondary structure analysis showed that H_N_519–845 retained majority of its alpha-helical secondary structure as in the native BoNT/A (Fig. 1C).

### Binding of H_N_519–845 to synaptosomes and synaptic vesicle

**Figure 2:**
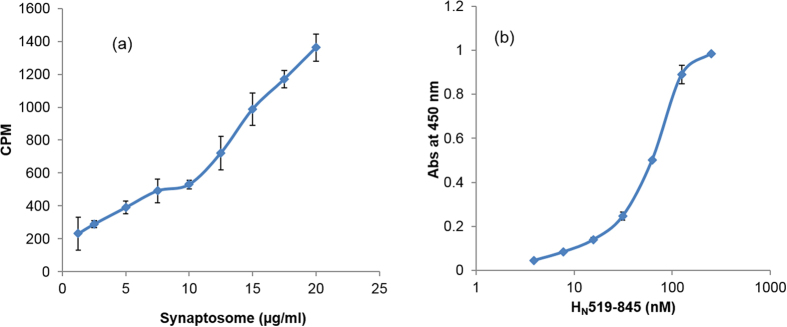
Binding of H_N_519–845 to mouse brain SNPs and SV. (**a**) Binding of peptide H_N_519–845 to SNPs was determined by incubating fixed amounts of ^125^I-labeled peptide (50,000 cpm) with increasing amounts of SNPs (1.25 to 20 μg/ml). The graph shows an increase in the binding of ^125^I-labeled H_N_519–845 with increased SNPs concentration. The assays were carried out in triplicates (n = 3 ± SD) (**b**) Binding of peptide H_N_519–845 to SV lysate was determined by adding different concentrations of H_N_519–845 (250–3.9 nM) to a SV-coated plate (10 μg/ml) and incubating the plate at 37 °C for 1 h. The plate was washed and the SV-H_N_ interaction was probed with mouse anti-his antibody followed by using horseradish peroxidase (HRP)-labelled anti-mouse antibody. An increase in the binding of H_N_519–845 was observed with its increasing concentration, finally becoming saturated at 250 nM concentration (n = 3 ± SD).

Assays using H_N_519–845 showed that the expressed peptide was capable of binding mouse brain synaptosomes (SNPs) and synaptic vesicle (SV). A solution phase assay was carried out using SNPs in which increasing concentrations of SNPs (1.25 to 20 μg/ml) were incubated with a fixed amount of peptide H_N_519–845. Figure 2a shows an increase in the binding of ^125^I-labeled peptide H_N_519–845 to increasing amounts of SNPs.

Similarly, a solid phase assay was carried out by plating out SV lysate and incubating varying amounts of H_N_519-845 (3.90–250 nM) with it. An increase in the binding of H_N_519–845 was observed with its increasing concentration (Fig. 2b).

### Preparation and characterization of anti-HN519–845 antibodies

**Figure 3:**
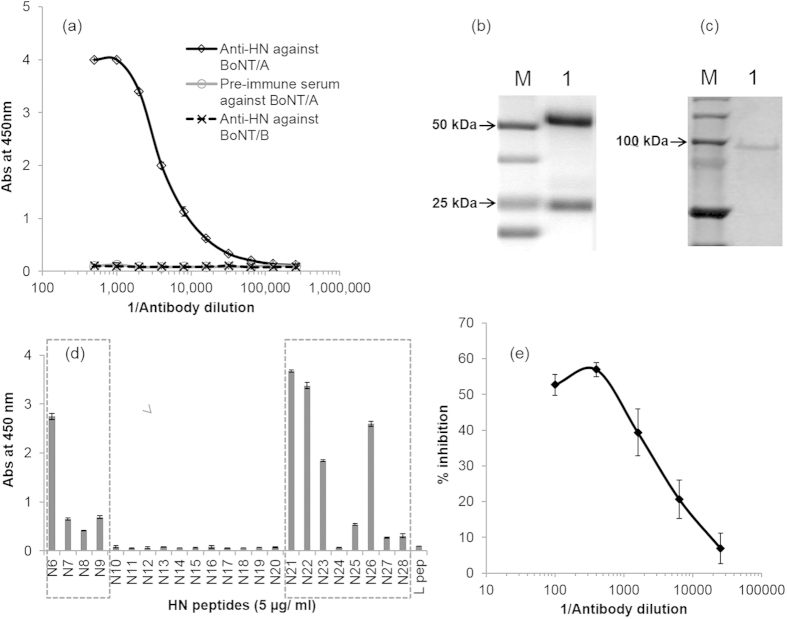
Characterization of mouse anti-H_N_519–845 antibodies. (**a**) Mouse anti-H_N_512–845 serum was titered against BoNT/A. The solid line represents the BoNT/A H_N_ specific antibody response obtained from 5 mice after 3 successive boosts with H_N_512–845 in Freund’s incomplete adjuvant. The broken line represents the binding of anti-H_N_512–845 serum to BoNT/B and the gray line represents the BoNT/A response obtained from the mice before primary immunization. A high titer (1/64,000) of anti-H_N_512–845 was obtained from the mice immunized with recombinant H_N_512–845 (n = 3 ± SD). (**b**) Purification of anti-H_N_512–845 pAb using protein G. Lane M, Bio-Rad Precision Plus Protein^TM^ Dual Xtra Standard; Lane 1, purified anti-H_N_512–845 pAb. (**c**) Immunoblotting of BoNT/A using anti-H_N_512-845 pAb. Lane M, Bio-Rad Precision Plus Protein TM Dual Xtra Standard; Lane 1, BoNT/A. (**d**) Antibody profiling of anti-H_N_512–845 pAb using synthetic H_N_ peptides. Synthetic peptides (19 amino acid each), spanning the H_N_512–845 region, were coated on a plate and the pAb (1/1000 dilution) was checked for binding the peptides. The binding was detected using HRP-labelled anti-mouse antibody. A mixture of peptides from the light chain region of BoNT/A was used as negative control. A high antibody response was observed against N6, N7 N21, N22, N23, and N26, whereas, a moderate to low antibody response was observed for peptides N8, N9, N25, N27 and N28. (**e**) An inhibition assay was carried out using anti-H_N_512–845 to check if the pAb can inhibit the binding of ^125^I- labelled BoNT/A to SNPs. A fixed concentration of ^125^I- labelled BoNT/A (50,000 cpm) was incubated (1 h) with different dilutions anti-H_N_512–845 pAb (1/100 to 1/25,600) and 10 μg of SNPs were added to the mixture and incubated for an additional 20 min. The assay showed that more than 50% inhibition of BoNT/A binding to the SNPs was obtained (n = 3 ± SD).

Pooled immune serum isolated by immunizing mice with H_N_519–845 provided a high antibody titer of 1/64,000 against BoNT/A (Fig. 3a). The Abs were specific to BoNT/A as indicated by absence of Abs binding to BoNT/B. Highly purified Abs (2 mg/ml) were obtained after protein G purification of 1 ml mice sera (Fig. 3b), which showed specific binding to the BoNT/A H-chain (Fig. 3c).

The epitope specificity of Abs were profiled using a solid-phase enzyme-linked immunosorbent assay (ELISA) assay using synthetic overlapping peptides (19 amino acid long with 5 amino acid overlapping regions) spanning H_N_519–845 region. Antibody responses were observed for peptides representing C and N-terminal regions of H_N_519–845 with high response against N6, N21, N22, N23, and N26, whereas, a moderate to low Ab response against N7, N8, N9, N25, N27 and N28 (Fig. 3d). Inhibition analysis using mouse anti-H_N_512–845 Abs showed that the Abs inhibited more than 50% of BoNT/A-SNP interaction (Fig. 3e), indicating the presence of blocking Abs.

### Mouse protection assay using peptides

**Figure 4:**
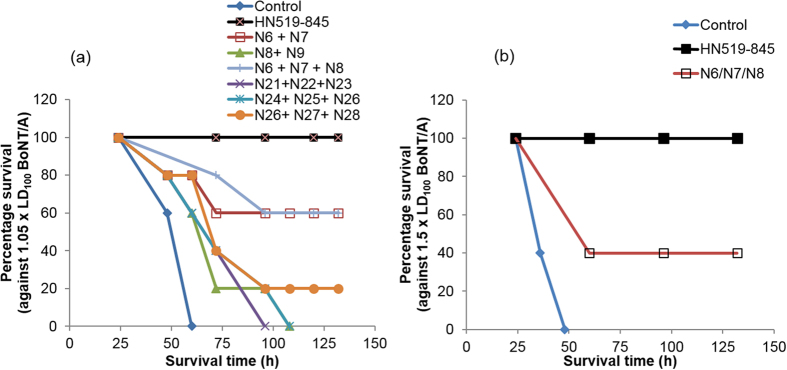
Characterization of peptide H_N_519–845 and corresponding synthetic peptides based on its protective efficacy against BoNT/A. (**a**) MPA was carried out by premixing H_N_519–845 (25 μg) and different combinations of synthetic peptide spanning the BoNT/A H_N_519–845 (7 μg each) with lethal dose of BoNT/A (1.05 × LD_100_). The toxin-peptide mixture was injected intravenously into 5 mice along with the controls, which were injected with BoNT/A (1.05 × LD100), without peptide. The mice were kept under observation for six days. The assay showed that premixing H_N_519–845 showed complete protection (100%) at 25 μg dose. The protection shown by H_N_519–845 was specific for BoNT/A as no protection was observed against BoNT/B at similar dose (data not shown). Combination of peptides N6, N7, N8 and 26, 27, 28 showed a partial protection 60% and 20%, respectively, indicating these regions may be important in generation of blocking antibodies. (**b**) MPA studies were repeated for H_N_519–845 and peptide combination N6, N7, N8 by using a slightly higher dose of BoNT/A (1.5 × LD_100)_ to confirm their protective efficacy. A complete protection (100%) was observed for H_N_519–845 N6, N7, N8 and a partial protection (40%) was observed for the synthetic peptides N6, N7, N8.

H_N_519–845 (25 μg) and combinations of synthetic peptides (7 μg), interacting with anti-H_N_519–845 Abs, were used to determine their protective efficacy *in vivo* against lethal dose of BoNT/A. H_N_519–845 showed a 100% protection, whereas, equimolar peptide mixtures of N6 + N7; N6 + N7 + N8; N8 + N9 and N26+ N27+ N28 showed a partial protection of 60%, 20% and 60%, 20%, respectively (Fig. 4a). The protection of H_N_519–845 and peptide mixture N6 + N7 + N8 was subsequently confirmed against 1.5 × LD_100_ BoNT/A, where they provided 100% and 40% protection, respectively (Fig. 4b).

### Mouse protection assay using anti-HN 519–845 antibodies

**Figure 5:**
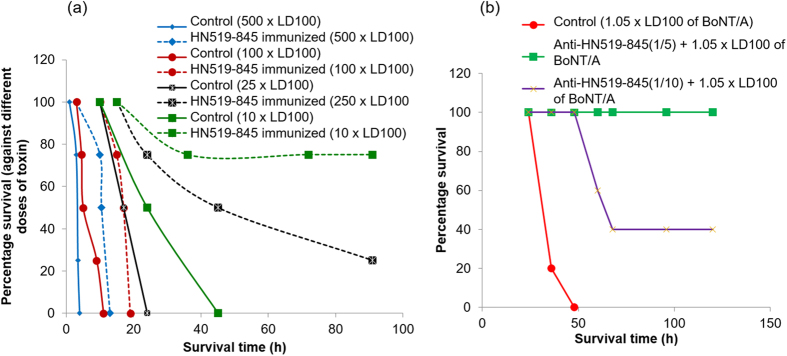
Evaluating protective efficacy of anti-H_N_519–845 by active and passive MPAs. (**a**) Active MPA analysis carried out by immunizing the mice intraperitoneally with H_N_519–845 and after two boosts challenging the mice with different doses of active BoNT/A. The mice were then compared with their respective un-immunized controls to evaluate protective efficacy of anti-H_N_519–845 generated. An increase in the survival time (~9 h) of mice challenged with 500 × LD_100_ and 100 × LD_100_ was observed, whereas, partial protection, 25% and 75%, were obtained at 25 × LD_100_ and 100 × LD_100_ BoNT/A challenges, thus, demonstrating that generated anti-H_N_519–845 are protective. (**b**) Passive MPA analysis carried out by incubating 1:5 and 1:10 dilution of anti-H_N_519–845 (from mice immunized with H_N_519–845) with 1.05 × LD_100_ BoNT/A and injecting them intravenously to another set of mice (5 mice each). Control mice were injected with toxin alone and used as reference for estimating protection by anti-H_N_519–845 sera. Anti-H_N_519–845 showed partial protection (40%) at 1:10 dilution, whereas complete protection (100%) was obtained at 1:5 dilution of anti-H_N_519–845 Abs.

Active and passive MPAs were carried out to evaluate the protective efficacy of anti-H_N_ Abs developed by immunizing the mice with H_N_519–845 region of BoNT/A. Active MPA showed that the immunized mice were partially protected (75% and 25%) at 10 and 25 × LD_100_ doses of BoNT/A with increase in the survival time at 100 and 500 × LD_100_ doses of BoNT/A (Fig. 5a). Passive MPAs were carried out using 1/5 and 1/10 dilutions of anti-H_N_519–845 sera. Anti-H_N_519–845 exhibited 100% protection against 1.05 × LD_100_ doses of BoNT/A at 1/5 dilution and 40% protection at 1/10 dilution showing that anti-H_N_519–845 can confer protection passively against active BoNT/A (Fig. 5b).

### Generation of H_N_519–593 and anti-H_N_729–845 antibodies

**Figure 6:**
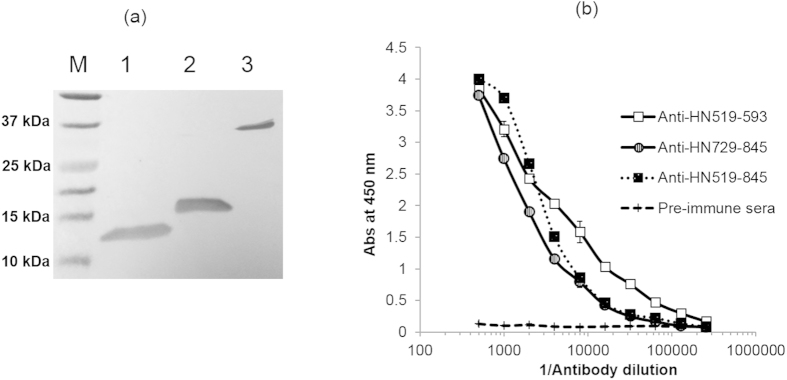
Expression of H_N_-fragments and their respective antibody generation. (**a**) Western blot analysis carried out using anti-H_N_519–845 sera (1/5,000 dilution). Lane M, Bio-Rad Precision Plus Protein TM Dual Xtra Standard; Lane 1, purified H_N_519–593; Lane 2, purified H_N_729–845 and lane 3, Lane 1, purified H_N_519–845; (**b**) Antibody titer from mice immunized with H_N_ peptides, H_N_519–593, H_N_729–845 and H_N_519–845, against active BoNT/A. A titer of >1:50,000 was obtained for both anti-H_N_519-845 and H_N_729–845, whereas, H_N_519–593 showed a high titer of >1:100,000 (n = 3 ± SD).

Abs developed against H_N_519–845 showed that a high antibody response was obtained against the N-terminal region (H_N_519–593) and C-terminal region (H_N_729–845) of H_N_519–845 (Fig. 3d). To analyze the regional contribution in development of blocking Abs, H_N_519–593 and H_N_729–845 were expressed and purified separately (Fig. 6a) followed by Ab generation in mice. High Ab titers were obtained against BoNT/A for both H_N_519–593 and H_N_729–845 immunized mice (Fig. 6b). Anti-H_N_729–845 was profiled using synthetic peptides and the response was compared with Abs generated against H_N_519–845 and active BoNT/A. Almost comparable profiles were obtained for anti-H_N_519–593, anti-H_N_729–845 and anti-H_N_519–845 representing high immunogenicity of these representative BoNT/A H_N_ regions (Table 1).

### Electrophysiology analysis to investigate inhibition of channel formation by anti-H_N_ antibodies

**Figure 7:**
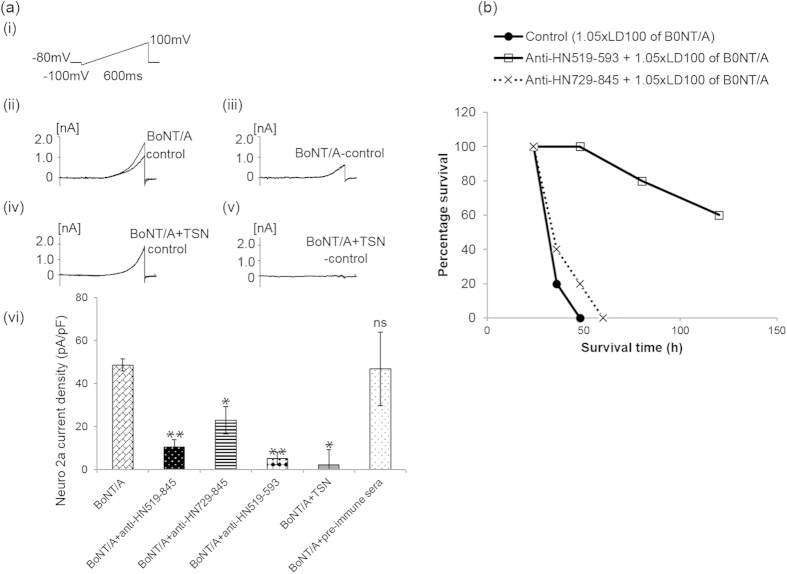
Evaluation of anti-H_N_-fragments in inhibiting BoNT/A-induced channel formation and protection. (**a**) Effect of BoNT/A and its antibodies on channel formation in neuro 2a cells by whole-cell patch clamp. (i) Voltage ramp protocol used in all assays. (ii) Representative current elicited in neuro 2a cells before (control) and after perfusing BoNT/A (4.16 μg/ml). (iii) Representative BoNT/A current after subtracting native neuro 2a cell currents. (iv) Representative current after adding BoNT/A with TSN. (v) Representative BoNT/A current in the presence of TSN after subtracting the native neuro 2a cell currents. (vi) Maximum current amplitude from the difference between control and test solutions plotted in the histogram as current density (pA/pF). Mann-Whitney U-test was done to compare the current density in control and the test solutions. *p < 0.05; **p < 0.01; ns = not significant (n = 3 to 5). Anti-H_N_519–593 and anti-H_N_519–845 showed maximum inhibition of channel formation by active BoNT/A, whereas, anti-H_N_729–845 showed only partial channel blockage. (**b**) Passive MPA analysis was carried with anti-H_N_519–593 and anti-H_N_729–845. The antisera were incubated with 1.05 × LD_100_ BoNT/A for 1 h and then administered intravenously to the mice. Control mice were injected with toxin (1.05 × LD_100_ BoNT/A) and used as reference for estimating protection by the antisera. Anti-H_N_519–593 showed partial protection (60%) at 1:1 dilution, whereas, no protection was obtained with anti-H_N_729–845 Abs.

Application of BoNT/A (4.16 μg/ml) resulted in channel formation in murine neuro 2a neuroblastoma cells (Fig. 7a). Channel formation was measured by patch-clamp electrophysiology in which the cells were held at −80 mV (Fig. 7a(i)). Outward currents were observed at positive voltages from 40 mV to 100 mV (Fig. 7a(ii)). Based on the composition of the pipet solution and the bath solution, the outward current is elicited predominantly by the efflux of K^+^ ions. This was confirmed using a non-specific K^+^  inhibitor TEA (1 mM) which blocked around 90% of the current (Fig. S1B).

Baseline current (control) was subtracted from BoNT/A-mediated channel opening to eliminate the contribution of endogenous neuro 2a currents (Fig. 7a(iii)). A known inhibitor of BoNT/A, toosendanin (TSN), was used as a positive control to prevent channel formation in neuro 2a cells by BoNT/A at 40 μM (Fig. 7a(iv and v)). Inhibition of BoNT/A-mediated channel formation by Abs against H_N_519–845, H_N_729–845 or H_N_519–593 was tested by incubating BoNT/A with Abs for 2 h at room temperature in external patch-clamp solution. BoNT/A incubated with anti-H_N_519–845 and anti-H_N_519–593 showed significant inhibition of channel opening in neuro 2a cells when compared to values with BoNT/A alone (p < 0.01) (Fig. 7a(vi)). Incubating BoNT/A with pre-immune sera did not affect BoNT/A-mediated channel formation and served as negative control for the analysis (Fig. 7a(vi)).

### Mouse protection assay using anti-H_N_519–593 and anti-H_N_ 729–845 antibodies

Passive MPAs were carried out using H_N_519–593 and H_N_729–845 specific Abs to determine the protective efficacy of these regions against lethal dose of BoNT/A (1.05 × LD_100_) BoNT/A. Anti-H_N_519–593 Abs showed 60% protection against BoNT/A, whereas, no protection was observed with anti-H_N_729–845 Abs against active BoNT/A (Fig. 7b).

## Discussion

Earlier vaccination against BoNT relied on formaldehyde-inactivated toxins (toxoids) and consisted of either monovalent (*i.e.* one) or pentavalent (five) toxoid serotypes. However, formaldehyde-treated toxins have adverse side effects. Determination of the sequences of BoNT genes has permitted construction of recombinant vaccines. Recombinant H_C_ of BoNT/A1, stimulated immune responses that protected mice against intraperitoneal toxin doses of up to 10^6^ LD_50_ of BoNT/A1^9^,^10^,^11^. The subdomains of the H_C_ domain (H_CN_ and H_CC_), and the catalytic L chain, or the L chain linked to the H_N_ domain (LH_N_) have been investigated. It was reported that the H_CN_ and H_CC_ domains of H_C_, when each used alone or as a mixture, stimulated lower protective immunity in mice than the intact H_C_ by itself^12^. Three monoclonal Abs (mAbs) prepared against BoNT/E L chain, H_N_ and H_C_ were reported to inhibit translocation of BoNTs A and E^13^. Mouse mAbs prepared^14^ after immunization with toxoid followed by BoNT/A recognized sites on the light chain, H_N_ and H_C_. A vaccine based on the L chain and the N-terminal half of the H chain (*i.e.* LH_N_) of toxins A and B has been tested^15^. To improve its protective capability, the LH_N_/A was cross-linked with formaldehyde. One injection of the cross-linked preparation protected mice against 103 × LD_50_ of BoNT/A1 and against BoNT/A subtypes A1, A2, and A3. Also, a single dose of LH_N_/B provided protection in mice against BoNT/B4 (non-proteolytic toxin subtype). However, the L chain by itself, or the L chain linked to the H_N_ domain (LH_N_) induced lower protective immunity than H_C_^16^. So the H_C_ domain seemed to be the smallest fragment for an optimal vaccine design. Intact whole toxins with mutations at the enzyme active site that detoxified the molecule have also been tested in mice as vaccine candidates and found to be effective in providing protective immunity against toxin poisoning^17^,^18^.

Using overlapping synthetic peptides spanning the entire BoNT/A and BoNT/B molecules, the antigenic sites were mapped^7^,^8^,^19^,^20^,^21^ with antisera from human volunteers immunized with a pentavalent toxoid containing BoNTs A, B, C, D and E, and with anti-toxoid antisera of horse, mouse and chicken. The sites that bind to mouse brain SNPs^1^,^22^ have also been mapped using overlapping synthetic peptides that covered the entire polypeptide chains of toxins A and B. It was found that regions on the H_C_ as well as regions on the H_N_ domains participate in the binding of the toxin to Abs and to SNPs. Region 785–803 was immunodominant with antisera of all four host species followed, in decreasing order, by regions 547–565, 743–761, 659–677 and much lower, but reproducible, amounts of Abs were bound, by some other H_N_ peptides. A recombinant peptide corresponding to region H_N_729–845 (H_N_729–845) of BoNT/A was found to bind directly to SNPs and to neuronal cells and to inhibit considerably the binding of BoNT/A to SNPs^2^. Significantly, the free peptide by itself protected mice against a lethal dose (1.05 × LD_100_) of BoNT/A and this compared well with the protection provided by free recombinant peptide 1163–1296 in the H_CC_ domain^2^, concluding that H_N_ in its free state can bind to the neuronal cells and inhibit toxin binding to the cell and prevent toxicity.

The epitopes of BoNTs A and B were also mapped^8^ with Abs from cervical dystonia patients who were treated with, and became immunoresistant to, native BoNT/A or BoNT/B. Anti-BoNT/A Abs recognized regions 785–803 (28 of 28 patients) and 743–761 (9 of 28 patients). Garcia-Rodriguez *et al.*^23^ obtained two mAbs from human volunteers immunized with a pentavalent toxoid similar to that mentioned above^7^. Using molecular evolution methods, they deduced that the mAbs bound to an epitope around residue 757 in the BoNT/A H_N_ domain, which falls within region 743–761 localized by Dolimbek *et al.*^8^. The two mAbs neutralized BoNTs A and B *in vivo* when pre*-*mixed with 200 × LD_50_ of toxin and injected intraperitoneally into 10 mice.

The aforementioned studies^8^ established that, upon immunization with the intact toxin, regions 785–803 and 743–761 of BoNT/A stimulated protecting Abs in humans and mice. These regions are located on the helical bundle of the H_N_ domain. But it was not known whether immunization with a suitable fragment carrying these regions would induce Abs that would bind to the same epitopes on the intact protein, and neutralize the toxin. Thus, in the present work, we carried out studies to determine the ability of a properly-selected fragment of H_N_ that carries the aforementioned epitopes to stimulate production of protective Abs against the toxin. If immune responses against an H_N_ fragment are found to be protective, then such a fragment, or perhaps active parts of it, could be incorporated into a synthetic vaccine design.

The present study identified the regions of BoNT/A H_N_ domain which are capable of producing blocking Abs that inhibit binding of the toxin to the cells and its toxicity. To achieve this, we cloned a peptide fragment corresponding to residues 519–845 of BoNT/A. This segment represents almost the entire anti-parallel helical bundle of H_N_. It retained much of its native folding in solution as confirmed by CD measurements indicating that intra- and inter-helical interactions were maintained in the segment’s solution folded structure. The radiolabeled peptide bound to SNPs and to SV lysate. Mouse Abs were generated against this fragment without cross-linking with formaldehyde and by a normal immunization procedure without optimizing the adjuvant. The Abs were characterized based on their submolecular specificity towards BoNT/A regions and also for their ability to inhibit BoNT/A binding to SNPs (Fig. 3e). The Abs were highly specific to the H-chain of BoNT/A (Fig. 3a,c), despite sharing a high sequence homology^24^ with BoNT/B. Immunodominant epitopes were identified on both N and C-terminal regions of H_N_519–845 compelling us to study these regions independently (Fig. 3d).

Initially, we studied H_N_519–845 by MPA using synthetic peptides spanning the entire segment. The study implied that N-terminal region of H_N_519–845, *i.e.* H_N_519–579, may be instrumental in the translocation process along with certain regions on the C-terminal part distributed randomly (Fig. 4a). Our previous study showed that H_N_729–845 can compete with BoNT/A and provide protection at low doses of BoNT/A^2^. To further investigate protection by the H_N_ domain, we expressed H_N_519–593 and H_N_729–845 individually and injected each in the mice to prepare Abs. The peptides exhibited no toxicity and produced Abs in high titers, recognizing both the peptides and BoNT/A. The Abs were compared to Abs against intact BoNT/A and against H_N_519–845, to identify the epitope specificity of each anti-peptide Ab (Table 1). Abs against each peptide bound equally well to the respective peptide and to the intact toxin, indicating that these peptides elicited Abs that were primarily directed against a native-like conformation of the BoNT/A (Table 1 and Fig. 6b).

Antibodies against peptides H_N_519–845 and H_N_519–593 significantly inhibited ion channel formation by BoNT/A on neuro 2a cell membrane, whereas Abs against peptide H_N_729–845 caused partial (about 50%) inhibition (Fig. 7a(vi)). Inhibition of channel formation can be either due to increased cargo size or limiting receptor availability^25^. During voltage ramp protocol, part of the outward current is contributed by endogenous ion channels in neuro 2a cells. Voltage-gated K^+^  channels such as Kv1.1, Kv1.4, Kv2.1 and some members of TASK channels, TASK1 and TASK2, were described in neuro 2a cells^26^,^27^. TEA, a non-specific K^+^  inhibitor, blocked the outward current by 90% suggesting most of the BoNT/A-mediated currents and endogenous channels-mediated currents are due to K^+^  efflux. Remainder could be contributed by Cl^−^ influx, dominant in the external solution. Additionally, in a step protocol from −100 to 70 mV we recorded a peak inward current at −10 mV similar to Na^+^  current^28^. However, the inward current was not detected in the ramp protocol and did not affect the BoNT/A mediated-outward current. Therefore inhibition of BoNT/A-mediated ion channels by Abs against H_N_ peptide regions represents inhibition of K^+^  efflux.

Electrophysiology data was supported by MPA analysis, where, antibodies against H_N_519–845 and H_N_519–593 were protective against lethal dose of BoNT/A. On the other hand, Abs against H_N_729–845 were not protective. Peptide H_N_519–845 contains all the region represented by H_N_729–845 but is 210 residues longer than H_N_729–845. Consequently, the specificities possessed by these two Ab responses were different as expected (Table 1). The antigenic sites recognized by the Abs against H_N_729–845 were also present on the H_N_519–845 but the latter recognized additional antigenic sites within residues 519–593. This indicated that the Ab responses against region 519–593 are important for protection against the whole toxin. This was in fact confirmed by the finding that Abs against H_N_519–593 exerted high (60%) protection against 1.05 × LD_100_ of BoNT/A. In addition, even when equimolar amounts of free peptides N6, N7, N8 and N9 were premixed with BoNT/A and the toxin-peptides mixture was injected into mice, it protected them against BoNT/A poisoning.

It is concluded that the H_N_ domain carries binding sites to the cell membrane and is capable of generating blocking (protective) Abs against BoNT/A. Antibodies against appropriate parts of H_N_ will block ion channels on the membrane and in the free state inhibit the toxicity of intact toxin *in vivo*. The present studies did not test different adjuvants or attempt to maximize/optimize the neutralizing Ab response. It is very hard to compare protective activity of anti-H_N_519–593 with that of anti-H_C_ Abs. Those studies^10^,^29^,^30^,^31^,^32^,^33^ measured toxin challenge doses in LD_50_ units, whereas the present work used toxin doses in 1.05 × LD_100_ units. We strongly believe that a construct comprising the two domains would provide a more efficient vaccine than either alone. Studies are in progress to compare H_N_519–593, H_C_ and the combination construct using the same immunization protocol and challenge dose for optimum vaccine design.

## Materials and Methods

### Ethics statement

The animal experiments described in the current study were approved by the Institutional Animal Care and Use Committee (IACUC) of Baylor College of Medicine (BCM) (Protocol Number: AN-3018) and carried out in accordance with the recommendations in the Guide for the Care and Use of Laboratory Animals of the National Institutes of Health.

### Reagents

Active BoNT/A and B were obtained from Metabiologics (Madison, WI) as a solution (0.25 mg/ml) in 0.15 M NaCl in 0.01 M phosphate buffer, pH 7.2 (PBS) containing 25% (v/v) glycerol and stored at −20 °C. Bacterial expression components: BL21(DE3)pLysS competent cells, pET-26b(+) vector and Terrific Broth (TB) were obtained from Invitrogen (Carlsbad, CA). All other chemicals were purchased from Sigma Aldrich (St. Louis, MO), unless otherwise stated.

### Cloning and expression of H_N_ peptides

BoNT/A heavy chain gene (Okra strain) was synthesized by GenScript (Piscataway, NJ). H_N_519–845, H_N_519–593 and H_N_729–845 were amplified from the BoNT/A heavy chain gene using primers H_N_519-845-for 5′- GGC CGG ATC CGA TGA ATC TTT CAA GTG ATA TTA TTG GTC AAT TAG AA-3′ and H_N_519-845-rev 5′-CCT TAG CGG CCG CAA GTG TAT TAT TAA CTT TAT CTT TTA AAC G-3′, H_N_519-593-for 5′- GGC CGG ATC CGA TGA ATC TTT CAA GTG ATA TTA TTG GTC AAT TAG AA-3′ and H_N_519-593-rev 5′- CCT TAG CGG CCG CTT TCT TTA CAT AGT CTG AAG A-3′ and H_N_729-845-for 5′-GGC CGG ATC CGA TGC GTA AAA AAA TGA AAG AAG C-3′ and H_N_729-845-rev 5′-CCT TAG CGG CCG CAA GTG TAT TAT TAA CTT TAT CTT TTA AAC G-3′. The amplicons were digested with *Bam*HI/*Not*I enzymes and cloned into the pET-26b(+) vector.

Positive clones were expressed in BL21(DE3)pLysS *E. coli* cells and purified from inclusion bodies, under denaturing conditions, using immobilized metal affinity chromatography (IMAC). The isolated protein was refolded and thoroughly buffer exchanged in phosphate buffered saline (PBS)^2^, followed by its qualitative and quantitative analysis by SDS-PAGE and BCA protein assay kit (Thermo Scientific, Rockford, IL).

### Synaptosome binding analysis

Isolation of SNPs from mouse brain was done as originally described^34^ with minor modifications^22^. Peptide was labeled with ^125^I (Perkin Elmer, Waltham, MA) by the chloramine T method^35^ and binding was analyzed as described by Ayyar *et al.*^2^. Briefly, a fixed amount of ^125^I-labeled peptide (50,000 cpm) was added to different concentrations of SNPs (1.25 to 20 μg/ml in 100 μl of Ringer’s buffer). After 20 min incubation at 37 °C SNPs were collected and washed three times with Ringer’s buffer and the bound radioactivity was measured in an automatic gamma counter (LKB-Wallac, Turku, Finland). The binding results were corrected for non-specific binding by subtracting the binding values of the controls.

### Synaptic vesicle binding analysis

Synaptic vesicles were purified from mouse brain as described by Huttner *et al.*^36^ with few modifications. Briefly, brains from 10 mice were homogenized in 10 volumes of sucrose buffer (320 mM sucrose, 5 mM HEPES-KOH (pH 7.6), 1 mM EDTA, protease inhibitor cocktail (Sigma Aldrich, St. Louis, MO) using glass-Teflon homogenizer. The homogenate was centrifuged for 10 min at 1000 × g and the pellet was resuspended in 0.64 M buffered sucrose. The suspension was centrifuged (45,000 × g) and the pellet was resuspended in PBS containing 2% Triton X-100 and protease inhibitor cocktail (Sigma Aldrich, St. Louis, MO) and stirred for 30 min on ice. The lysate was clarified by centrifugation (45,000 × g), aliquoted and stored in −80 °C.

Peptide-SV binding was analyzed by ELISA by serially diluting the peptide in 1% bovine serum albumin (BSA) (w/v) in PBS containing 0.5% (v/v) Tween^®^20 (PBST) and adding the dilutions (100  μl ) to MaxiSorp™ ELISA plate coated with SV lysate (10 μg/ml), blocked with 3% BSA (w/v) in PBS. The plate was incubated at 37 ^o^C for 1 h and washed 3 × 3 with PBST and PBS. The binding was detected using mouse anti-His mAb (Thermo Scientific, Rockford, IL) diluted 1/2000 in 1% (w/v) BSA-PBST and subsequently probing the reaction with 1/2000 dilution of HRP-labelled anti-mouse IgG (Fc-specific) (Sigma Aldrich, St. Louis, MO) in 1% (w/v) BSA-PBST. The reaction was detected by adding 100 μl of 3,3′,5,5′-tetramethylbenzidine (TMB) substrate for 10 min followed by quenching with 50 μl of 10% HCl. The plate was read at 450 nm absorbance and the background (wells without SV or H_N_519–845) was subtracted.

### Production of mouse antibodies against H_N_ peptides

Female ICR mice (18 to 22 g) were immunized with 5 μg of peptides in a 1:1 emulsion of PBS and Freund’s complete adjuvant (FCA) administered by subcutaneous injection. The mice were boosted with 2 μg H_N_ peptides in a 1:1 emulsion of peptide in PBS and Freund’s incomplete adjuvant (FICA) on day 14 and 28. Blood was collected from the immunized mice on day 40, and sera was harvested, pooled and stored at −80 ^o^C.

### Determination of mouse anti-H_N_ antibody titer

Antibody titer of mice H_N_729–845 immune sera was determined by preparing serial dilutions of antisera (1/500 to 1/2,56,000) in 1% (w/v) BSA and titrating it against 0.5 μg/ml of BoNT/A in a solid-phase ELISA. The BoNT/A-specific IgG component was detected with HRP-labelled anti-mouse IgG (Fc-specific) antibody (1/2000 in 1% BSA (w/v) in PBST) followed by developing the reaction with TMB substrate.

### Synaptosome inhibition analysis

Synaptosome inhibition analysis was carried out by incubating different dilutions (1/100 to 1/25,600) of Ab with ^125^I-labeled BoNT/A (50,000 cpm), for 1 h at 37 ^o^C. A fixed amount (1 μg) of SNPs was added to the Ab-BoNT/A mixture and incubated for 20 min at 37 ^o^C (100 μl final reaction volume). SNPs were collected, washed and bound radioactivity was measured. The percent inhibition of ^125^I-labeled BoNT/A binding was calculated relative to uninhibited controls.

### Antibody purification and specificity analysis

Antibodies were purified by diluting 1 ml of mice anti-H_N_ serum in 10 ml PBS. Diluted serum was passed through a column containing 1 ml of PBS-equilibrated protein G resin (GenScript, Piscataway, NJ). The column was washed with 30 ml PBS and the bound IgG was isolated by passing 1 ml of 0.1 M glycine, pH 2.2. The isolated Ab fraction was neutralized by adding 1 ml of 1 M Tris-HCl, pH 8.0, and buffer exchanged in PBS.

Immunoblotting analysis was carried out by resolving BoNT/A (5 μg) on a SDS-PAGE gel and transferring the protein to a nitrocellulose membrane. The membrane was blocked with 10% blocking grade milk (Bio-Rad, Hercules, CA) and subsequently incubated with 1/10,000 dilution of purified anti-H_N_ Ab for 1.5 h at room temperature. The membrane was washed 3 × 3 with PBST and PBS and BoNT/A-Ab binding was detected with HRP-labelled anti-mouse IgG (Fc-specific) Ab.

### Mapping of antibody epitopes using synthetic H_N_ peptides

A MaxiSorp™ ELISA plate was coated with 100 μl of 5 μg/ml synthetic peptides^1^ spanning the H_N_ region. The plate was incubated 1 h at 37 °C and blocked with 3% BSA (w/v) in PBS. Hundred μl of purified anti-H_N_ Abs (diluted 1/1000 in 1% (w/v) BSA) was added to the plate and incubated for 1 h at 37 °C. The interaction of peptide with specific IgG component was detected by incubating the wells with HRP-labelled anti-mouse IgG (Fc-specific) Ab (1/2000 in 1% BSA (w/v) in PBST) followed by detecting the reaction with 100 μl of TMB-substrate. The analysis was carried out in triplicates for each peptide and a mixture of BoNT/A light chain peptides was used as control in this study.

### Determining *in vivo* protective efficacy of H_N_ peptides

LD_100_ of the active BoNT/A preparation was determined in ICR outbred mice, by injecting different doses (5 mice per dose) of the toxin intravenously and intraperitoneally. The mice were observed 3 times a day for six days. The lowest dose at which no mice survived was defined as LD_100_. The protective efficacy of the peptides against BoNT/A was then investigated by mouse protection assays (MPAs). Active BoNT/A (1.05 × LD_100_) was mixed with H_N_519–845 (25 μg) and with various combinations of peptides (5 μg each), showing Ab titer on H_N_519–845 immunizations (N6+N7, N8+N9, N6+N7+N8, N21+N22+N23, N24+N25+N26 and N26+N+N28) right before injection. The mixtures were then injected intravenously (5 mice per dose). A control group received only 1.05 × LD_100_ of active BoNT/A without any peptide. The mice were observed 3 times a day for six days. Protective efficacy of the H_N_519–845 and peptides N6+N7+N8 was further assessed by administering the peptides with increased lethal dose of BoNT/A (1.5 × LD_100_).

### Determination of *in vivo* protective efficacy of mouse anti-H_N_ antibodies

Active and passive MPAs were carried out using anti-H_N_519–845 Abs to analyze the blocking Abs in the generated repertoire. Active MPA was carried out by immunizing the mice with a 1:1 ratio of 5 μg of H_N_519–845 and FCA intraperitoneally. The mice were boosted 2 times at 2 weeks intervals by 2 μg of H_N_519–845 in FICA. Bleed was collected 10 days after the second boost and serum was harvested and tittered against BoNT/A. Immunized mice were challenged after a week with different doses of active BoNT/A. Unimmunized mice were used as controls for each dose of the toxin.

For passive MPA, sera containing anti-H_N_ Abs (anti-H_N_519–845, anti-H_N_519–593 and H_N_729–845) were mixed with active BoNT/A (1.05 × LD_100_) at 1:5 and 1:10 dilutions and incubated for 1 h at 37 °C. The serum-toxin mixture was injected intravenously (5 mice per dose) with control group receiving only 1.05 × LD_100_ of active BoNT/A. The mice were observed 3 times a day for six days and protective efficacy of anti-H_N_519–845 Ab was evaluated by referencing them with the control group.

### Patch-clamp analysis

Whole-cell patch-clamp studies were performed using an automated Port-a-patch setup (Nanion, North Brunswick, NJ) connected to a HEKA EPC 10 USB amplifier as described previously^37^. Patch chips had a resistance of 2–4 MΩ with internal solution containing 145 mM KF, 10 mM HEPES, 10 mM EGTA, 2 mM MgCl_2_ at pH 7.2 and 290 mOsm. The external solution contained 160 mM NaCl, 4.5 mM KCl, 2 mM CaCl_2_, 1 mM MgCl_2_, 10 mM HEPES at pH 7.2 and 300 mOsm. A voltage ramp protocol was applied to the cells in whole-cell configuration by applying −100 mV to 100 mV for 600 ms while holding the cell at −80 mV. The external solution was exchanged for test solutions containing the toxin and/or Abs in the bath chamber. Current density was recorded before and after this exchange and plotted in a bar graph. To assess the efficacy of each antibody in reducing channel formation, the current density measured in the presence of BoNT/A alone was compared to that measured in the presence of BoNT/A and each of the different antibodies using multiple Mann-Whitney U-tests.

## Additional Information

**How to cite this article**: Ayyar, B.V. *et al.* Antigenic sites on the H_N_ domain of botulinum neurotoxin A stimulate protective antibody responses against active toxin. *Sci. Rep.* **5**, 15776; doi: 10.1038/srep15776 (2015).

## Supplementary Material

Supplementary Information

## Figures and Tables

**Table 1:** Summary of site specificity of mouse antibodies against BoNT/A and the three H_N_ peptides H_N_519–845, H_N_729–845 and H_N_519–593*.

Peptide	Sequence	Abs againstBoNT/A	Abs againstPeptide	Abs againstPeptide	Abs againstPeptide
Number	Position	Toxoid	H_N_519–845	H_N_729–845	H_N_519–593
N5	505–523	−			
N6	519–537	+++	++++		+++++
N7	533–551	+++	+++		++++
N8	547–565	+++++	++		++
N9	561–579	++++	++		+++++
N10	575–593	+	−		+
N11	589–607	−	−		
N12	603–621	−	−		
N13	617–635	−	−		
N14	631–649	±	−		
N15	645–663	−	−		
N16	659–677	−	−		
N17	673–691	±	−		
N18	687–705	−	−		
N19	701–719	+	−		
N20	715–733	±	−		
N21	729–747	−	+++++	++++	
N22	743–761	+	+++++	+	
N23	757–775	−	+++	++	
N24	771–789	+	−	±	
N25	785–803	+++++	++	−	
N26	799–817	−	++++	+++	
N27	813–831	+++	+	+	
N28	827–845	+++	+	±	
N29	841–859	−			

^*^The number of + signs indicates the strength of antibody binding to the respective peptide.
