# Supplementary material for: Antigenic sites on the HN domain of botulinum neurotoxin A stimulate protective antibody responses against active toxin
Source: Sci Rep. 2015 Oct 28;5:15776. doi: 10.1038/srep15776 (PMC4623786; doi:10.1038/srep15776)
Supplement: Supplementary Information [file srep15776-s1.pdf]

Supplementary Figure

For *Scientific Reports*

Antigenic sites on the H_N domain of botulinum neurotoxin A stimulate protective antibody responses against active toxin

B. Vijayalakshmi Ayyar[1], Rajeev B. Tajhya[2], Christine Beeton[2] and M. Zouhair Atassi[1,3,*]

[1] Department of Biochemistry and Molecular Biology, [2] Department of Molecular Physiology and Biophysics, [3] Department of Pathology and Immunology, Baylor College of Medicine, Houston, Texas 77030, USA

*** Corresponding author:**

M. Zouhair Atassi, PhD, DSc
Robert A. Welch Chair of Chemistry
Department of Biochemistry and Molecular Biology
Baylor College of Medicine
Houston, Texas 77030
Tel: (713) 798-6050
E-mail: matassi@bcm.edu

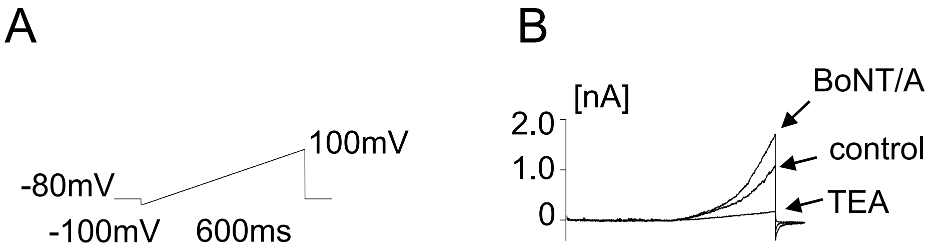

Figure S1. Effect of BoNT/A and a K^+ channel blocker on pore formation in neuro 2a neuroblastoma cells by whole-cell patch clamp. (A) Voltage ramp protocol used in all assays. (B) Representative current in neuro 2a cells (control) after addition of BoNT/A (4.16 µg/ml) followed by addition of 1 mM tetraethyl ammonium (TEA).
